# mTOR Signaling Upregulates CDC6 via Suppressing miR-3178 and Promotes the Loading of DNA Replication Helicase

**DOI:** 10.1038/s41598-019-46052-8

**Published:** 2019-07-08

**Authors:** Xianjin Wu, Shenghua Li, Xing Hu, Xiaoliang Xiang, Megan Halloran, Linlin Yang, Terence M. Williams, Peter J. Houghton, Changxian Shen, Zhengfu He

**Affiliations:** 1 Key Laboratory of Hunan Province for Study and Utilization of Ethnic Medicinal Plant Resources, College of Biological and Food Engineering, Huaihua University, Huaihua, 418008 China; 2 Comprehensive Cancer Center, The Ohio State University, Columbus, Ohio 43210 USA; 3 The Greehey Children’s Cancer Research Institute, The University of Texas Health Science Center at San Antonio, San Antonio, Texas 78229 USA; 4 Department of Thoracic Surgery, Sir Run Run Shaw Hospital, College of Medicine Zhejiang University, Hangzhou, 310016 China

**Keywords:** Oncogenes, Checkpoint signalling

## Abstract

mTOR signaling pathway is deregulated in most cancers and uncontrolled cell cycle progression is a hallmark of cancer cell. However, the precise molecular mechanisms of the regulation of DNA replication and chromatin metabolism by mTOR signaling are largely unknown. We herein report that mTOR signaling promotes the loading of MCM2-7 helicase onto chromatin and upregulates DNA replication licensing factor CDC6. Pharmacological inhibition of mTOR kinase resulted in CHK1 checkpoint activation and decreased MCM2-7 replication helicase and PCNA associated with chromatins. Further pharmacological and genetic studies demonstrated CDC6 is positively controlled by mTORC1-S6K1 and mTORC2 signaling. miRNA screening revealed mTOR signaling suppresses miR-3178 thereby upregulating CDC6. Analysis of TCGA data found that CDC6 is overexpressed in most cancers and associates with the poor survival of cancer patients. Our findings suggest that mTOR signaling may control DNA replication origin licensing and replisome stability thereby cell cycle progression through CDC6 regulation.

## Introduction

Tumorigenesis is driven by gain-of-function mutations of oncogenes and loss-of-function alteration of tumor suppressor genes^1^. Chromosomal instability is a hallmark of cancer cell and contributes to chromosome translocation, aneuploidy, gene dosage change and other chromosomal chaos of cancer cells^2–4^. More than 100 years ago, Theodor Boveri proposed that chromosomal instability drives tumorigenesis. Recent data demonstrate that chromosomal instability drives a mutator phenotype both in yeast and human cancer cells^3,5,6^. It has been thought that inactivation of spindle assembly checkpoint (SAC) (also called mitotic checkpoint) leads to chromosomal instability; however, inactivation mutations of SAC components are not commonly found in human cancers^4^. Therefore, the molecular mechanisms underlying cancer cell chromosomal instability warrant further exploration.

DNA replication origin activation is crucial for maintaining genomic integrity in all organisms and is tightly regulated to occur only once per cell cycle^3^. A pre-replicative complex (pre-RC) forms at the origin of replication during late mitosis. Formation of pre-RC is a very important step for the complete and faithful duplication of the genome to ensure that each daughter cell will carry the same genetic information as the parent cell. In most eukaryotes a pre-RC is composed of six origin recognition complex proteins (ORC1-6), CDC6, CDT1 and a heterohexamer of MCM proteins (MCM2-7). CDC6 is the licensing factor of DNA replication and its deregulation results in impaired DNA replication thereby genome instability. CDC6 plays multiple roles in ensuring precise chromosome duplication^7^. CDC6 is not only required for replication origin licensing, but is also crucial for proper S-phase DNA replication progression^8^. CDC6 can trigger a checkpoint response, which could ensure that all DNA is replicated before mitotic entry^9^. Overexpression of replication initiation factors CDT1 and CDC6 along with cyclin A-CDK2 promotes re-replication in human cancer cells with inactive p53 but not in cells with functional p53^10^. Overexpression of CDC6 leads to suppression of INK4/ARF expression. Moreover, elevated level of CDC6 activates the Rad3-dependent checkpoint in fission yeast^11^. The most recent TCGA RNA-seq data reveal that CDC6 is overexpressed in most cancers, highlighting the importance of CDC6 deregulation in tumorigenesis. However, the underlying mechanism of CDC6 upregulation in cancer cells is unclear.

Cyclin dependent kinases (CDKs) coordinate cell cycle progression and is required for the processing of damaged DNA ends and checkpoint activation^12–14^. KRAS and PIK3CA are among the most commonly mutated genes across cancer types^15^. PI3K-AKT and RAS-MAPK are the predominant growth-promoting signaling pathways, which enhance CDKs activity and increase cell cycle progression. Of note, both pathways converge on mTOR signaling. mTOR kinase is a conserved member of the PI3K-related kinase family. mTOR lies at the hub of intracellular and extracellular signal transduction networks via integrating and processing multiple signals, and dictates the rates of macromolecule synthesis and hence cell growth, proliferation, metabolism and survival^16–18^. Mounting data have demonstrated that mTOR signaling deregulation leads to cancers^16,19,20^. Recent data indicate that mTOR signaling modulates DNA damage response and hence DNA damage repair, providing an explanation for the fact that inhibiting RAS/PI3K-mTOR pathway sensitizes a lot of cancer cells to chemotherapy and radiotherapy^21–26^. Most components of DNA replication machineries are overexpressed in the majority of cancers that are associated with the poor overall survival (OS) and/or disease free survival (DFS) of lots of cancer patients (TCGA data). However, whether deregulation of mTOR signaling contributes to the upregulation of DNA replication machineries leading to replisome instability remains to be determined.

In the present study, we used rhabdomyosarcoma RH30 cell line as a model to explore the role and mechanisms of mTOR signaling on the control of DNA replication origin licensing. Our results demonstrate that mTOR signaling promotes DNA replication origin licensing thereby cell cycle progression through upregulating CDC6.

## Materials and Methods

### Chemicals and reagents

Rapamycin, AZD8055 and MK2206 were from Selleck Chemicals (Houston, TX). Mimics and inhibitors of miR-3178 were from Applied Biosystems. Nocodazole was purchased from Sigma (St. Louis, MO). Subcellular protein fractionation kit was purchased from Thermo Scientific.

### Cells, siRNA and plasmids

Rh30, Rh18, Rh41, HeLa, WI-38, NB-1691 and NB-1643 cells were cultured in RMPI 1640 (GIBCO) supplemented with 10% heat-inactivated FBS (GIBCO). 4E-BP1/2 double knock out MEFs were from Nahum Sonenberg (McGill University, Canada). S6K1, S6K2 and S6K1/2 single and double knock out MEFs were provided by George Thomas (University of Cincinnati). RICTOR knockout MEFs were from Mark A. Magnuson (National Institute of Neuroscience, Japan). MEFs were cultured in DMEM (GIBCO) supplemented with 10% heat-inactivated FBS (GIBCO). Control and ON-TARGETplusSMARTpool siRNAs of mTOR were purchased from Dharmacon (Chicago, IL). Plasmid pcDNA3 Myr-AKT1 was a gift of William Sellers (Addgene plasmid 9008). Plasmid pcDNA-CDC6 was from John Diffley (Cancer Research UK London Research Institute). Plasmid pCMV-Akt1-wt was from Said Sebti (Moffitt Cancer Center). Lipofectamine 2000 was from Invitrogen (Carlsbad, CA).

### Immunoblotting

Cells were lysed on ice in RIPA lysis buffer (Cell Signaling Technology) supplemented with protease and phosphatase inhibitors (Roche), and 1 mM PMSF (Sigma). Immunoblots were probed with the following antibodies: S6, pS6 (S235/236), AKT, pAKT (S473), pTOBP1-S1159, pATM (S1981), pCHK2 (T68), S6K1, pS6K1 (T89), γH2AX, cleaved Caspase 3, GAPDH, CDC6, MCM2, MCM3, MCM7, CDT1, PCNA, β-Actin, CDC25A, RB, pRB (S780), CHK1, pCHK1 (S345), pCHK1 (S280). All antibodies were from Cell Signaling Technology.

### Real-time quantitative RT-PCR

Total RNA or microRNA from cultured cells were extracted with mirVana miRNA Isolation Kit (Ambion) according to the manufacturer’s instructions. Reverse transcription of mRNA was performed using the High Capacity RNA-to-cDNA kit (Applied Biosystems). TaqMan MicroRNA Reverse Transcription Kit was used to produce cDNA for TaqMan MicroRNA Assay (Applied Biosystems). Real-time PCR was performed on the 7900HT Fast Real-Time PCR System (Applied Biosystems) using the TaqMan® Universal Mastermix (Applied Biosystems). Human and mouse CDC6 expression was quantified in real-time with CDC6-specific FAM dye-labeled MGB-probes and normalized to GAPDH (Applied Biosystems). Human miR-3178 was quantified in real-time with FAM dye-labeled MGB-probes and normalized to RNU66 or snU6 (Applied Biosystems).

### miRNA profiling

Rh30 cells were treated with DMSO or 0.5 μM AZD8055 for 24 hr, total RNAs were extracted for miRNAs profiling with a miRNA array by LC Sciences, LLC (Houston, TX, USA). Data were analyzed by LC Sciences, LLC and the miRNAs alteration with p < 0.05 were plotted. The targets of the significantly altered miRNAs were analyzed by the online TargetScan and miRDB programs.

### Statistical analyses

Graphs were constructed using GraphPad Prism (Graphpad Software, San Diego, CA). All data are presented as mean ± SEM. Statistical significance was determined by unpaired two-tailed *t* tests or two-way ANOVAs. *P < *0.05 was considered statistically significant.

## Results

### Transient inhibition of mTOR kinase results in CHK1 phosphorylation at S345

**Figure 1:**
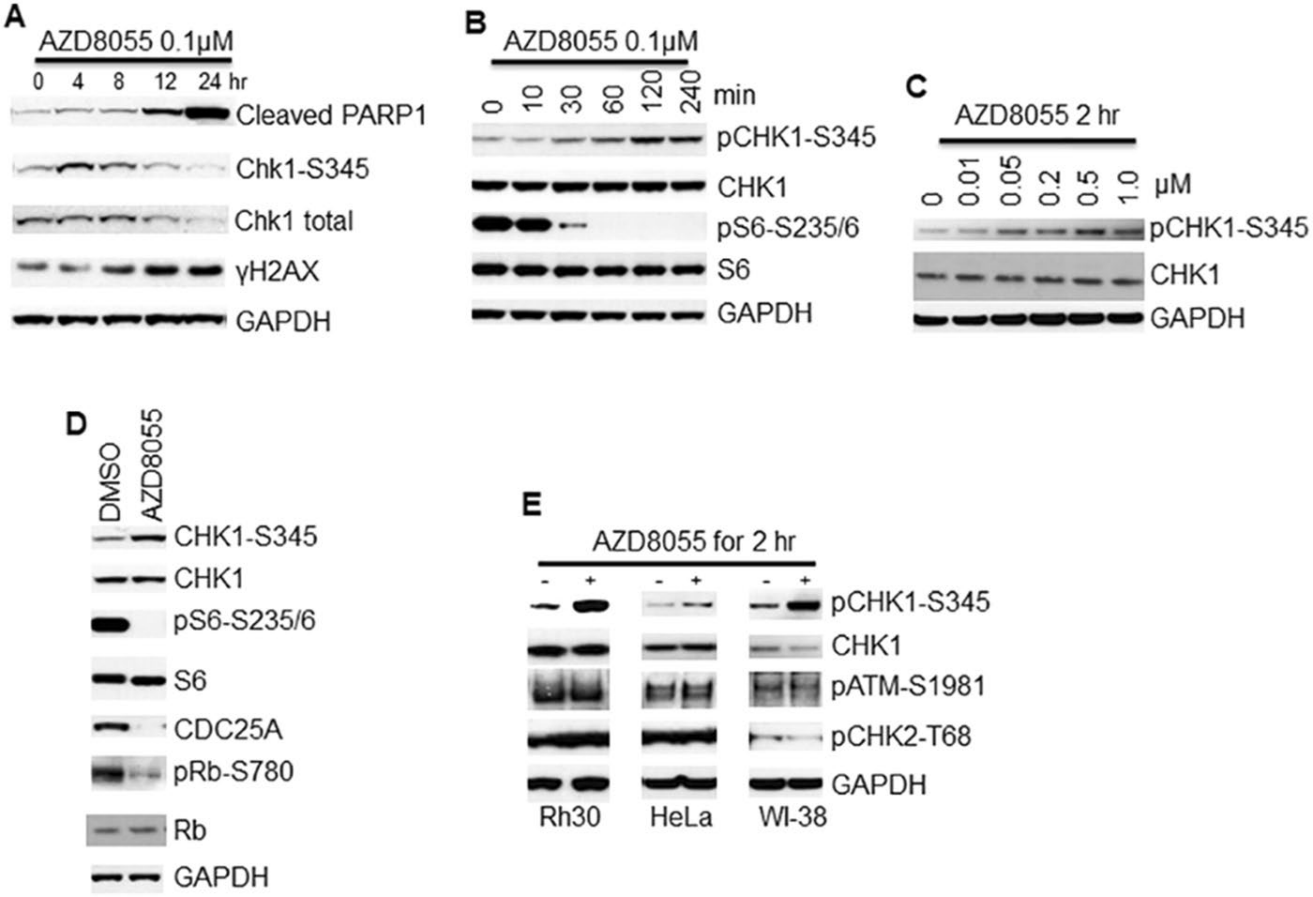
Transient inhibition of mTOR kinase results in CHK1 checkpoint activation. (**A**,**B**) Rh30 cells were treated with 0.1 μM AZD8055 for the indicated time and total proteins were extracted for immunoblotting of the indicated proteins with GAPDH as loading control. (**C**) Rh30 cells were treated with the indicated concentrations of AZD8055 for 2 hr. Total proteins were extracted for immunoblotting of CHK1 and pCHK1-S345 with GAPDH as loading control. (**D**) Rh30 cells were treated with DMSO or 0.1 μM AZD8055 for 2 hr and subjected immunoblotting of the indicated proteins. (**E**) Rh30, HeLa and WI-38 cells were treated with 0.1 μM AZD8055 for 2 hr. Total proteins were extracted for immunoblotting of pCHK1-S345, total CHK1, pATM-S1981 and pCHK2-T68 with GAPDH as loading control. Immunoblots were converted to white and black with auto tone by Photshop program.

During our pharmacodynamic studies of mTOR kinase specific inhibitor AZD8055 in rhabdomyosarcomas, we find that treatment of rhabdomyosarcoma Rh30 cells with AZD8055 leads to downregulation of CHK1 protein, which is accompanied by DNA damage as demonstrated by increased phosphorylation of H2AX (γH2AX) and apoptosis as shown by cleavage of PARP1 (Fig. 1A). Unexpectedly, we revealed a dynamic regulation of the phosphorylation of CHK1 (pCHK1) at S345 by AZD8055: AZD8055 induces phosphorylation of CHK1 without phosphorylation of H2AX and cleavage of PARP1 before 8 hr (Fig. 1A). To further explore this dynamic regulation of CHK1 activity by mTOR, we treated Rh30 and Rh18 cells with AZD8055 and determined pCHK1 at 0, 0.5, 1, 2, 4 and 8 hr. AZD8055 induced apparent pCHK1-S345 as early as 1 hr in both Rh18 and Rh30 cells (Figs 1B and S1A). At 0.5 hr, AZD8055 potently suppresses the activity of both mTORC1 and mTORC2 as demonstrated by the disappearance of pAKT-S473, pS6K1-T389 and pS6-S235/236 (Fig. S1A). Treatment of Rh30 cells with different concentrations of AZD8055 showed that AZD8055 induced pCHK1-S345 at 50 nM (Fig. 1C). Further immunofluorescence analysis revealed that AZD8055 treatment resulted in accumulation of pCHK-S345 foci (Fig. S1B). Activated CHK1 phosphorylates CDC25A leading to CDC25A degradation^27^. Consistently, treatment of Rh30 cells with 0.1 μM AZD8055 for 2 hr resulted in dramatic reduction of CDC25A and pRB-S780 (Fig. 1D). However, treatment of Rh30 cells with 0.1 μM AZD8055 for 2 hr did not result in phosphorylation of ATM at S1981 and CHK2 at T68. Similar results were observed in HeLa and WI-38 cells (Fig. 1E).

### Inhibition of mTOR kinase leads to dislodgement of MCM2-7 and PCNA from chromatin

**Figure 2:**
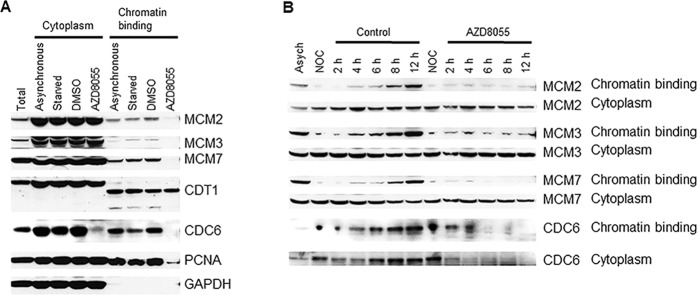
mTOR is essential for the maintenance and loading of MCM2-7 on chromatins. (**A**) Rh30 cells were serum starved for 24 hr, released to complete medium for 6 hr, then treated with AZD8055 (0.1 μM) for 2 hr, and subjected to subcellular protein fractionation and immunblotting. (**B**) Rh30 cells were arrested in prometaphase by 50 ng/ml nocodazole for 24 hr, released to complete medium or medium containing AZD8055 (0.1 μM) for 2, 4, 6, 8 and 12 hr. At each time point, the cells were harvested and subjected to subcellular protein fractionation and immunbloting. Immunoblots were converted to white and black with auto tone by Photshop program.

Uncoupling of DNA replication helicase MCM2-7 complex from replisome results in accumulation of ssDNA, which is rapidly coated by RPA leading to CHK1 activation^28^. To explore the potential mechanisms of CHK1 activation by AZD8055, Rh30 cells were serum starved for 24 hr to synchronize cells in arrested state, released to complete medium for 6 hr, and then treated with AZD8055 (0.1 μM) for 2 hr. The cells were fractioned into cytoplasm and chromatin binding parts. MCM2, MCM3, MCM7, CDT1, CDC6 and PCNA were evaluated by immunoblotting in each subcellular fraction. Intriguingly, we found dramatic reduction of MCM2, MCM3, MCM7 and PCNA in chromatin binding fraction but not cytoplasm fraction of cells treated with AZD8055. There is no apparent alteration of CDT1 in all fractions with or without AZD8055 treatment. Intriguingly, CDC6 was dramatically reduced both in the cytoplasm and chromatin-binding fractions of the cells treated with AZD8055 (Fig. 2A). In order to explore whether mTOR kinase is required for the loading of MCM2-7 complex, we arrested Rh30 cells at prometaphase with 50 ng/ml nocodazole for 24 hr, and then released them to complete medium or medium containing AZD8055 for 2, 4, 6, 8 and 12 hr. Cell fractionation assay showed that there was no chromatin binding of MCM2, MCM3, and MCM7 during prometaphase arrest by nocodazole, and MCM2, MCM3 and MCM7 proteins were gradually loaded onto chromatin in normal medium after release from nocodazole arrest. In sharp contrast, AZD8055 obviously prevented the loading of MCM2, MCM3 and MCM7 proteins onto chromatins even after 12 hr, while AZD8055 did not change the levels of MCM2, MCM3 and MCM7 in the cytoplasm. There was apparent chromatin binding of CDC6 during prometaphase arrest by nocodazole and CDC6 was increasingly loaded onto chromatin when the cells were released into normal medium; whereas chromatin bound CDC6 was gradually lost when the cells were released into the medium containing AZD8055 (Fig. 2B). These results suggest a critical role for mTOR in the maintenance and loading of MCM2-7 complex on chromatins.

### mTOR positively regulates CDC6

**Figure 3:**
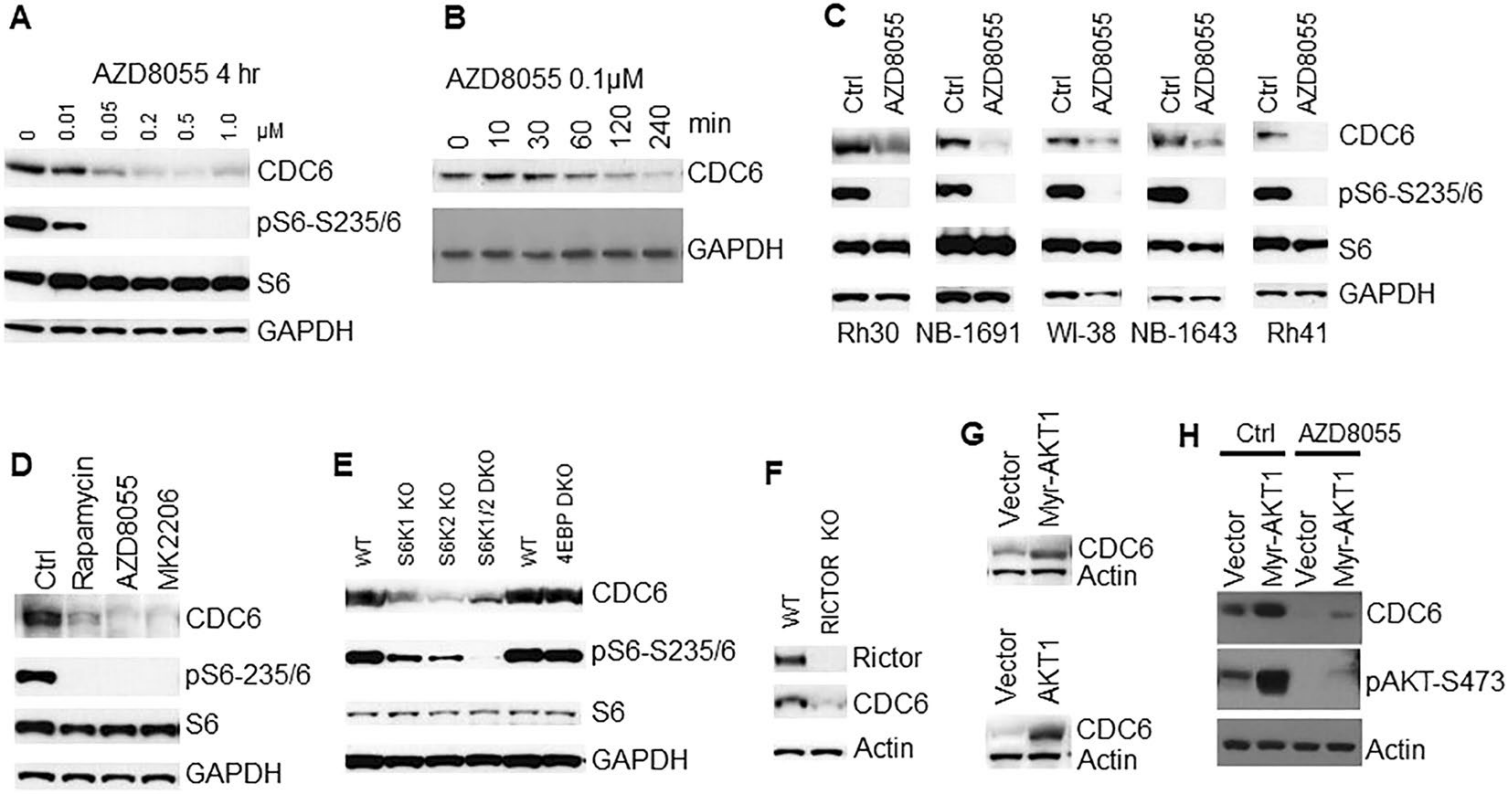
mTOR positively regulates CDC6. (**A**) Immunoblotting of CDC6 in Rh30 cells treated with the indicated concentrations of AZD8055 for 4 hr. (**B**) Immunoblotting of Rh30 cells treated with 0.1 μM AZD8055 for the indicated time. (**C**) Different cancer cells were treated with 0.1 μM AZD8055 for 4 hr. CDC6 protein level was assessed by immunoblotting. (**D)** Immunoblotting Rh30 cells treated with 100 nM rapamycin, 0.1 μM AZD8055 or 1 μM MK2206 for 4 hr. (**E**) Immunoblotting analysis of exponentially growing wild type (WT), S6K1 KO, S6K2 KO, S6K1/2 double KO, and 4E-BP1/2 double KO MEFs. (**F**) Exponentially growing wild type (WT) and RICTOR KO MEFs were subjected to immunoblotting analysis of CDC6. (**G**) Rh30 cells were transfected with empty vector, plasmid pAKT1, or plasmid of Myr-AKT1 for 48 hr, followed by immunoblotting analysis of CDC6. (**H**) Rh30 cells were transfected with empty vector or plasmid of Myr-AKT1 for 48 hr, followed by treatment with 0.1 μM AZD8055 for 4 hr. CDC6 protein level and pAKT-S473 were assessed by immunoblotting. Immunoblots were converted to white and black with auto tone by Photshop program.

CDC6 is essential for the loading of MCM2-7 complex during cell cycle. The dramatic reduction of CDC6 in both cytoplasm and chromatins following mTOR inhibition suggests that mTOR may promote the loading and maintenance MCM2-7 complex on chromatins by positively regulating CDC6. To test the regulation of CDC6 by mTOR signaling, Rh30 cells were treated with increasing concentrations of AZD8055 for 4 hr. Immunoblotting of CDC6 protein level showed AZD8055 reduced CDC6 at 50 nM (Fig. 3A). When Rh30 cells were treated with 0.1 μM AZD8055 for different time, we found that CDC6 protein level was reduced as early as 1 hr (Fig. 3B). We next treated NB-1691, WI-38, NB-1643 and Rh41 cells in parallel with Rh30 cells with 0.1 μM AZD8055 for 4 hr, and found CDC6 was reduced by AZD8055 in all tested cell lines (Fig. 3C). To assess whether regulation of CDC6 by mTOR depends on mTORC1 and/or mTORC2, Rh30 cells were treated with 100 nM rapamycin (mTORC1 inhibitor), 0.1 μM AZD8055 (both mTORC1 and mTORC2 inhibitor) or 1 μM AKT kinase inhibitor MK2206 for 4 hr. Immunoblotting of CDC6 showed all the drugs decreased CDC6 (Fig. 3D). S6K1 and 4E-BP1 are the key downstream targets of mTORC1. We therefore assessed CDC6 in S6K1 knockout (KO), S6K2 KO, S6K1/2 double KO, and 4E-BP1/2 double KO MEFs, and found that depletion of S6K1, S6K2 or both S6K1 and S6K2, but not 4E-BP1/2, led to reduction of CDC6 (Fig. 3E). Consistent with the pharmacological inhibition results, RICTOR knockout MEFs had reduced CDC6 (Fig. 3F), while Rh30 cells transfected with plasmids pAKT1 or Myr-AKT1 demonstrated increased CDC6 (Fig. 3G). However, AZD8055 still potently reduced CDC6 in Rh30 cells transfected with myr-AKT1 plasmid, probably due to the strong inhibition of pAKT-S473 even under over-activation of AKT signaling (Fig. 3G). Thus mTOR kinase regulates CDC6 via both mTORC1 and mTORC2 signaling.

### mTOR suppresses miR-3178

**Figure 4:**
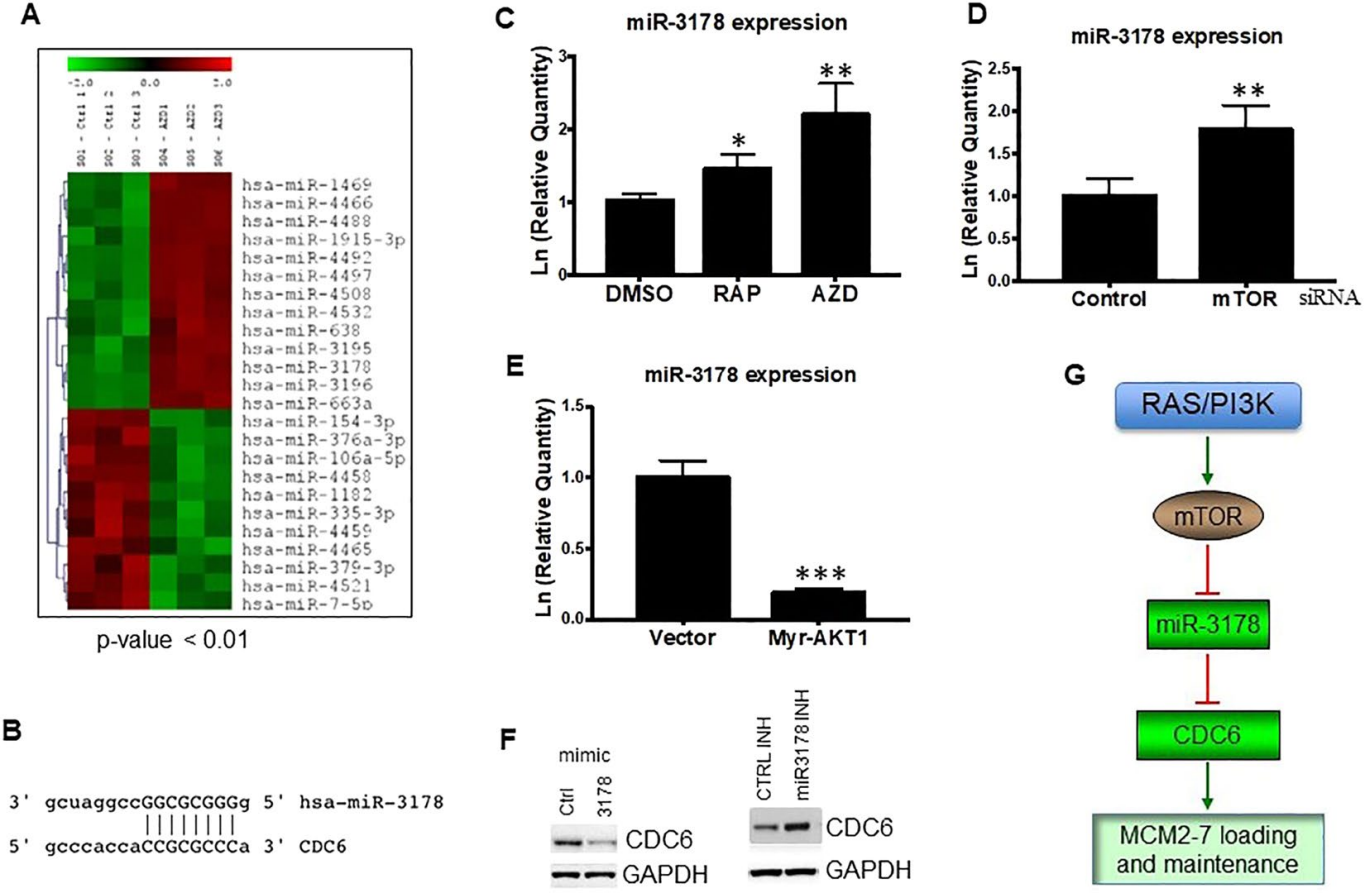
mTOR downregulates miR-3178. (**A**) Rh30 cells were treated with 0.1 μM AZD8055 for 24 hr, total RNAs were extracted for miRNAs profiling with a miRNA array. The miRNAs alteration with p < 0.01 were plotted. (**B**) The 3’UTR of CDC6 mRNA has a miR-3178 binding seed sequence. (**C**) RT-PCR analysis of miR-3178 in Rh30 cells treated with rapamycin or AZD8055. *p < 0.05, *p < 0.01 vs DMSO. (**D**) RT-PCR analysis of miR-3178 in Rh30 cells transfected with 100 nM scrambled control or mTOR siRNA. *p < 0.01 vs Control. (**E**) RT-PCR analysis of miR-3178 in Rh30 cells transfected with empty vector or plasmid of Myr-AKT1. *p < 0.001 vs Vector. (**F**) Immunoblotting of CDC6 in Rh30 cells transfected with 100 nM scrambled control, or miR-3178 mimics or inhibitor for 48 hr. Immunoblots were converted to white and black with auto tone by Photshop program. (**G**) Schema of the regulation of CDC6 by mTOR signaling as revealed by this study.

Recent advances have shown that more than 80% of human genome is transcribed and most of the transcripts are non-coding RNAs including long non-coding RNAs (lncRNAs) and microRNAs (miRNAs), which play a critical role in the regulation of chromatin dynamics^29,30^. To explore the potential mechanism of the promotion of cell cycle progression by mTOR signaling, Rh30 cells were treated with 0.1 μM AZD8055 for 24 hr, total RNAs were extracted for profiling of miRNAs with a miRNA array. We found 54 miRNAs were significantly downregulated while 42 significantly upregulated by AZD8055 (P < 0.05, Figs 4A and S2). Analysis of some targets of the miRNAs regulated by mTOR revealed that miR-3178 and miR-26a, both of which target CDC6 (Fig. 4B), were dramatically unregulated by AZD8055, providing a potential mechanism of the regulation of CDC6 by mTOR. It has been demonstrated that miR-26a inhibits DNA replication licensing of lung cancer^31^ and ovarian cancer cells^32^ by targeting CDC6. MiR-3178 was found to be downregulated in hepatocellular carcinoma tumor endothelial cells^33^ and during lymphatic metastasis of gastric cancer^34^. Moreover, among the altered miRNAs following AZD8055 treatment, miR-3178 is one of the top upregulated mRNAs. Therefore, in the present study, we focused on miR-3178. To validate the upregulation of miR-3178 by mTOR inhibition as screened from miRNA array analysis, Rh30 cells were treated with 0.1 μM rapamycin or 0.1 μM AZD8055 for 4 hr. RT-PCR analysis showed that both rapamcyin and AZD8055 significantly increased miR-3178 (Fig. 4C). Consistently, silencing of mTOR by siRNA significantly increased miR-3178 (Fig. 4D). In contrast, Rh30 cells transfected with plasmids of Myr-AKT1 significantly decreased miR-3178 (Fig. 4E). To assess the targeting of CDC6 by miR-3178, Rh30 cells were transfected with 100 nM scrambled control, or miR-3178 mimics or inhibitor for 48 hr. CDC6 protein level was assessed by immunoblotting, the results showed that miR-3178 mimics suppressed while miR-3178 inhibitor increased CDC6 protein levels (Fig. 4F).

## Discussion

The RAS-RAF-MEK-ERK and PI3K-AKT-mTOR pathways promote the growth, proliferation and survival of cells and are frequently altered in human cancers. Thus targeting the nodes of these two pathways is a key therapeutic strategy. However, the clinic outcome of the agents targeting these nodes as monotherapy is disappointing^35–39^. Most importantly, targeting RAS-RAF-MEK-ERK leads to compensatory activation of PI3K-AKT-mTOR signaling^37,39^. This highlights the critical role for mTOR signaling in drug resistance and the urgent need to identify and validate alternative functions of mTOR in order to improve the outcome of targeted cancer therapies^40^. In this study, we revealed that inhibition of mTOR kinase resulted in dislodgement of MCM2-7 replication helicase and PCNA from chromatin, which was accompanied with dramatic increase of miR-3178 and reduction of its target CDC6. Our findings suggest that deregulation of mTOR signaling may promote DNA replication through upregulating CDC6 (Fig. 4G).

To our knowledge, this is the first study to dissect mTOR kinase in the modulation of DNA replication origin licensing factors. Cancer is a disease resulting from combined genetic and epigenetic alterations^1,41^. Recent advance has clearly demonstrated that there is an elevated level of DNA damage in most cancer cells, especially late-stage and relapsed cancers^42^. It has been proposed that this enhanced spontaneous DNA damage results from oncogenic signaling induced DNA replication stress^2^. How rapid proliferating cancer cells with high levels of DNA damage survive the potent genome surveillance systems is an open unresolved question in cancer biology^2^. The discovery of the involvement of mTOR in DNA replication origin licensing may provide the basis to further elucidate this outstanding enigma.

The ultimate reason of cancer is the errors derived from DNA replication and repair. Once fired, DNA replication proceeds with no return. Uncontrolled DNA replication origin firing leads to DNA replication stress which activates ATR/CHK1 replication checkpoint to inhibit DNA replication late origin firing and arrest cell cycle progression. Maintained mTORC1 signaling potentially may promote the adaptation of cancer cells to DNA replication stress leading to the genome instability and heterogeneity of cancer cells. mTOR signaling may promote the survival of cancer cells via enhancing the expression of FANCD2, CHK1 and RRM2 through CDK4/6 while inhibiting ATM, revealing the potential mechanisms of the increased cancer cell growth and proliferation under stressful conditions^21,23,24,26^. The discovery of mTOR dependent maintenance and loading of DNA replication components such as MCM2-7 and PCNA on chromatins might shed light on the mechanisms of oncogene-induced DNA replication stress.

It has been well-documented that mTOR is required for the progression of G1 phase, especially early G1 phase both in yeast and mammalian cells^16,18^. The discovery of the promotion of MCM complex loading onto chromatins by mTOR may disclose a novel mechanism of the regulation of the progression of M and G1 phases by mTOR signaling.

It has been shown that DNA damage leads to CDC6 decrease^43^. It was also reported that CDC6 overexpression triggers cell cycle checkpoints activation^9^. However, it remains unknown whether CDC6 overexpression results in DNA damage in human cancer cells. We found that overexpression of CDC6 resulted in slightly increase of pChk1-S345 in Rh30 cells (Fig. S3A). However, AZD8055 only partially reduced CDC6 and did not increase pChk1-S345 signaling in Rh30 cells transfected with pcDNA-CDC6 (Fig. S3B). It will be important to investigate whether the threshold of CDC6 protein level controls cell growth and proliferation.

CDC6 overexpression is associated with the poor survival of lots of cancer patients (Fig. S4). Our discovery that miR-3178 is suppressed by mTOR and targets CDC6 may provide one of the mechanisms of the deregulation of CDC6 in cancers. In addition, once and only once per cell cycle for each DNA replication origin activation is crucial for maintaining genome integrity^1,44^. The discovery of deregulation of pre-RC component CDC6 by mTOR signaling might reveal the mechanism of the promotion of genome instability and heterogeneity of cancer cells by the PI3K signaling pathway. Since mTOR signaling is upregulated in most cancers, the negative control of miR-3178 by mTOR signaling suggests that miR-3178 may be a novel tumor suppressor by targeting CDC6. Regarding the essential role for CDC6 in replication origin licensing, alteration of miR-3178 may impact on the maintenance and loading of MCM2-7, thereby cell cycle progression. CDC6 overexpression in most cancers suggests targeting CDC6 is a promising strategy for cancer therapies. However, no enzyme activity of CDC6 has yet been identified, therefore target CDC6 itself is formidable. miR-3178 attenuation was found to be associated with chemoresistance of breast cancer patients^45^. Regarding negative regulation of CDC6 by miR-3178 and therapeutic potential of miRNAs^30^, it is plausible to target CDC6 by replenishing miR-3178.

In summary, we demonstrated mTOR signaling promotes the loading of MCM2-7 helicase onto chromatins and upregulates CDC6 through suppressing miR-318. Our findings expand our understanding of mTOR signaling in tumorigenesis and CDC6 might represent a new target worthy for testing of therapeutic intervention.

## Supplementary information

Supplementary Figures
