# Supplementary material for: mTOR Signaling Upregulates CDC6 via Suppressing miR-3178 and Promotes the Loading of DNA Replication Helicase
Source: Sci Rep. 2019 Jul 8;9:9805. doi: 10.1038/s41598-019-46052-8 (PMC6614418; doi:10.1038/s41598-019-46052-8)
Supplement: Supplementary file 1 — Supplementary Figures [file 41598_2019_46052_MOESM1_ESM.doc]

SUPPLEMENTARY INFORMATION

mTOR Signaling Upregulates CDC6 via Suppressing miR-3178 and Promotes the Loading of DNA Replication Helicase

Xianjin Wu, Shenghua Li, Xing Hu, Xiaoliang Xiang, Megan Halloran, Linlin Yang, Terence M. Williams, Peter J. Houghton, Changxian Shen & Zhengfu He

**Supplementary Figures**

**
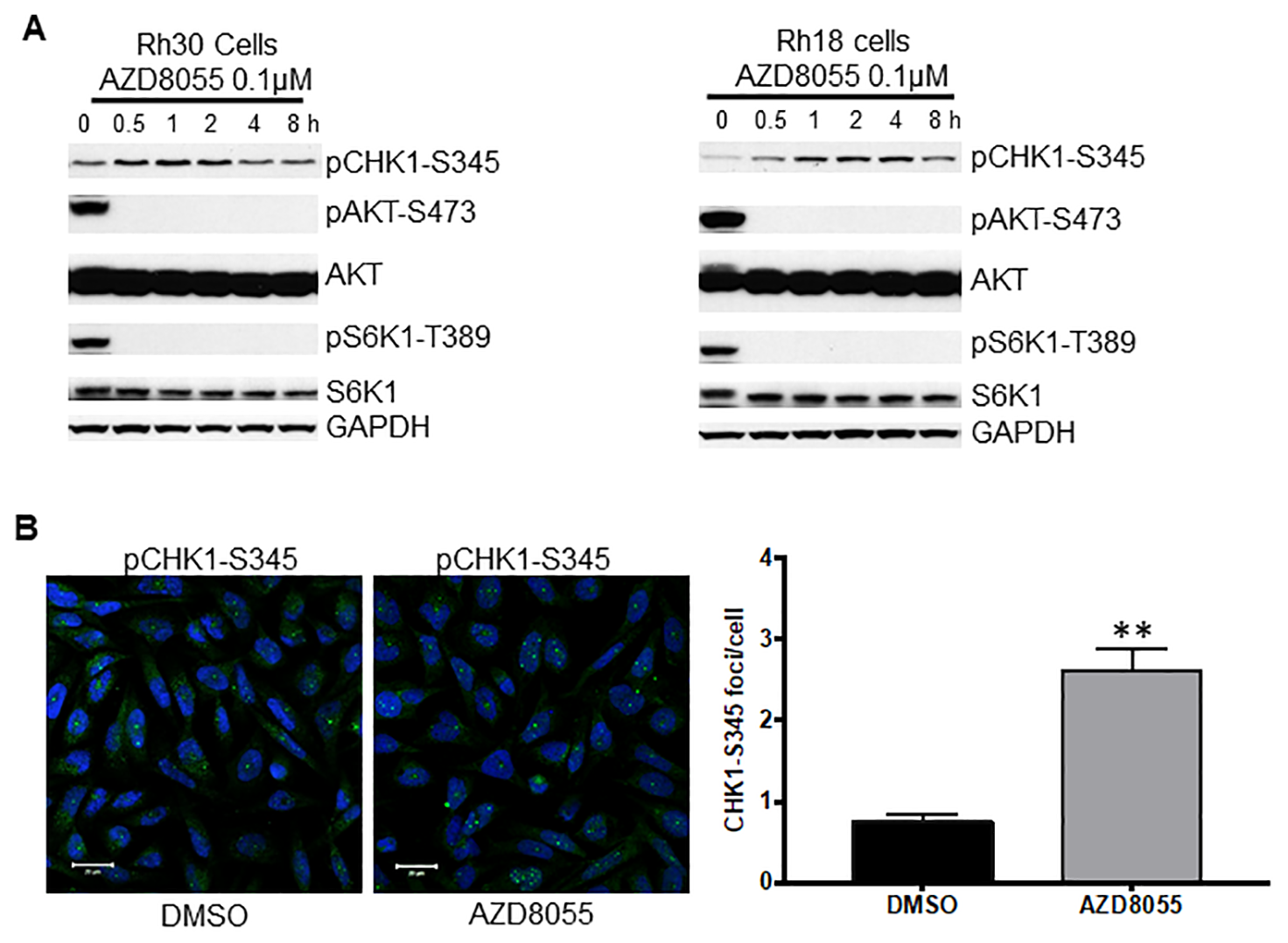
**

**Figure S1**. Transient inhibition of mTOR kinase increases pCHK1-S345 foci in nuclei, related to Figure 1. (**A**) Rh18 and Rh30 cells were treated with 0.1μM AZD8055 for the indicated time and total proteins were extracted for immunoblotting of pCHK1-S345, pS6K1-T389, total S6K1, pAKT-S473, and total AKT with GAPDH as loading control. Immunoblots were converted to white and black with auto tone by Photshop program. (**B**) Rh30 cells were treated with 0.1 μM AZD8055 for 2 hr and subjected to immunoflurescence staining of pCHK1-S345. Nuclei were stained with DAPI. 30 cells were counted in triplicate. **p<0.01 vs DMSO.

**
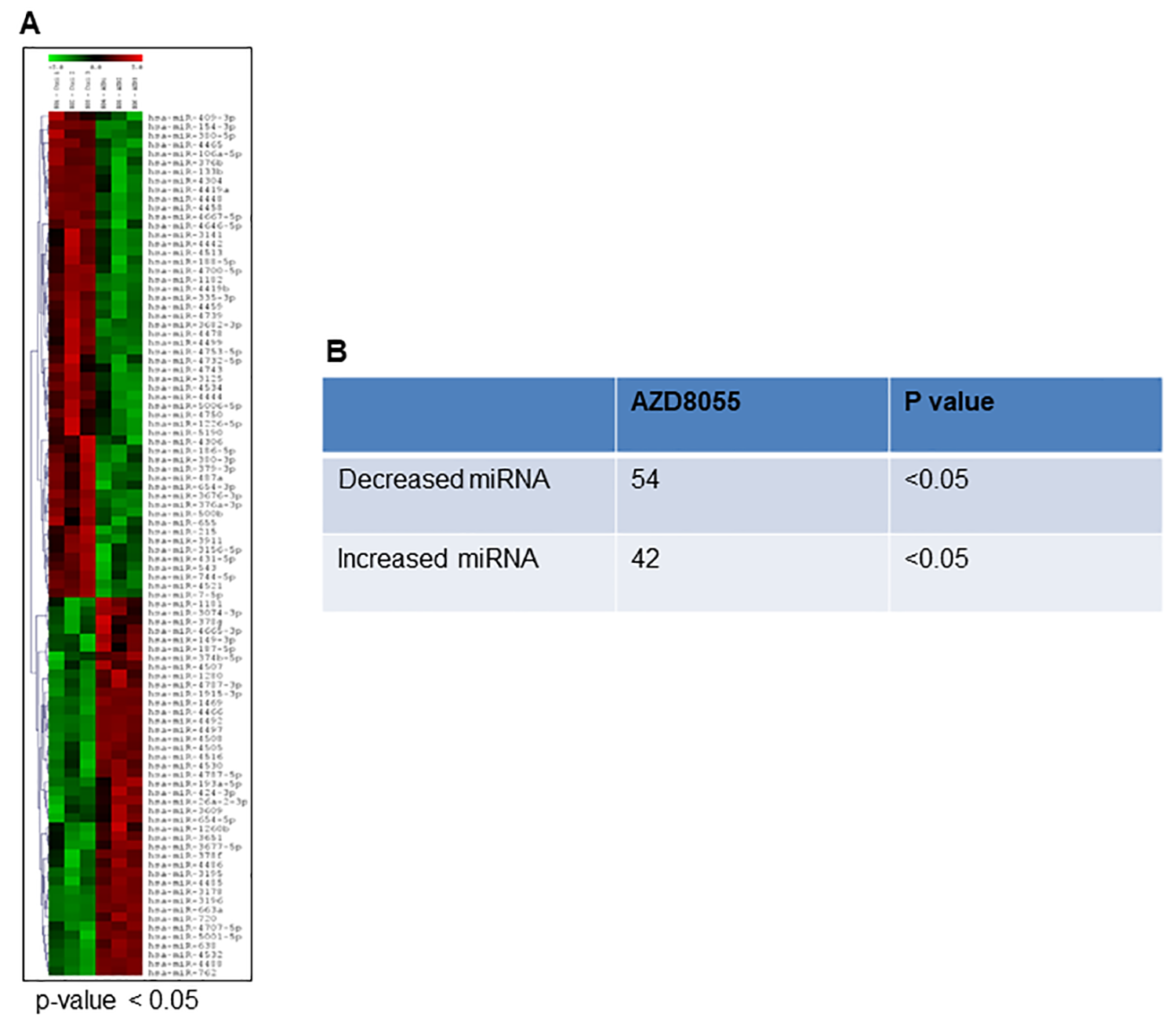
**

Figure S2. mTOR may regulate DNA replication via miRNAs, related to Figure 4A. (A) Rh30 cells were treated with 0.1μM AZD8055 for 24 hr, total RNAs were extracted for miRNAs profiling with a miRNA array. The miRNAs alteration with p<0.05 were plotted. (B) The miRNAs significantly upregulated and downregulated by AZD8055 were counted.


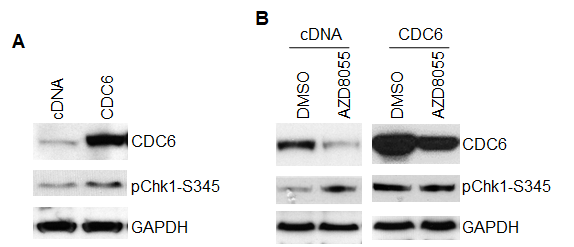


**Figure S3. CDC6 protein levels may contribute to CHK1 activation.** (**A**) Rh30 cells were transfected with empty vector pcDNA (cDNA) or plasmid of pcDNA-CDC6 (CDC6) for 48hr. CDC6 protein level and pChk1-S345 were assessed by immunoblotting. (**B**) Rh30 cells were transfected with empty vector pcDNA (cDNA) or plasmid of pcDNA-CDC6 (CDC6) for 48hr, followed by treatment with 0.1 μM AZD8055 for 4 hr. CDC6 protein level and pChk1-S345 were assessed by immunoblotting.


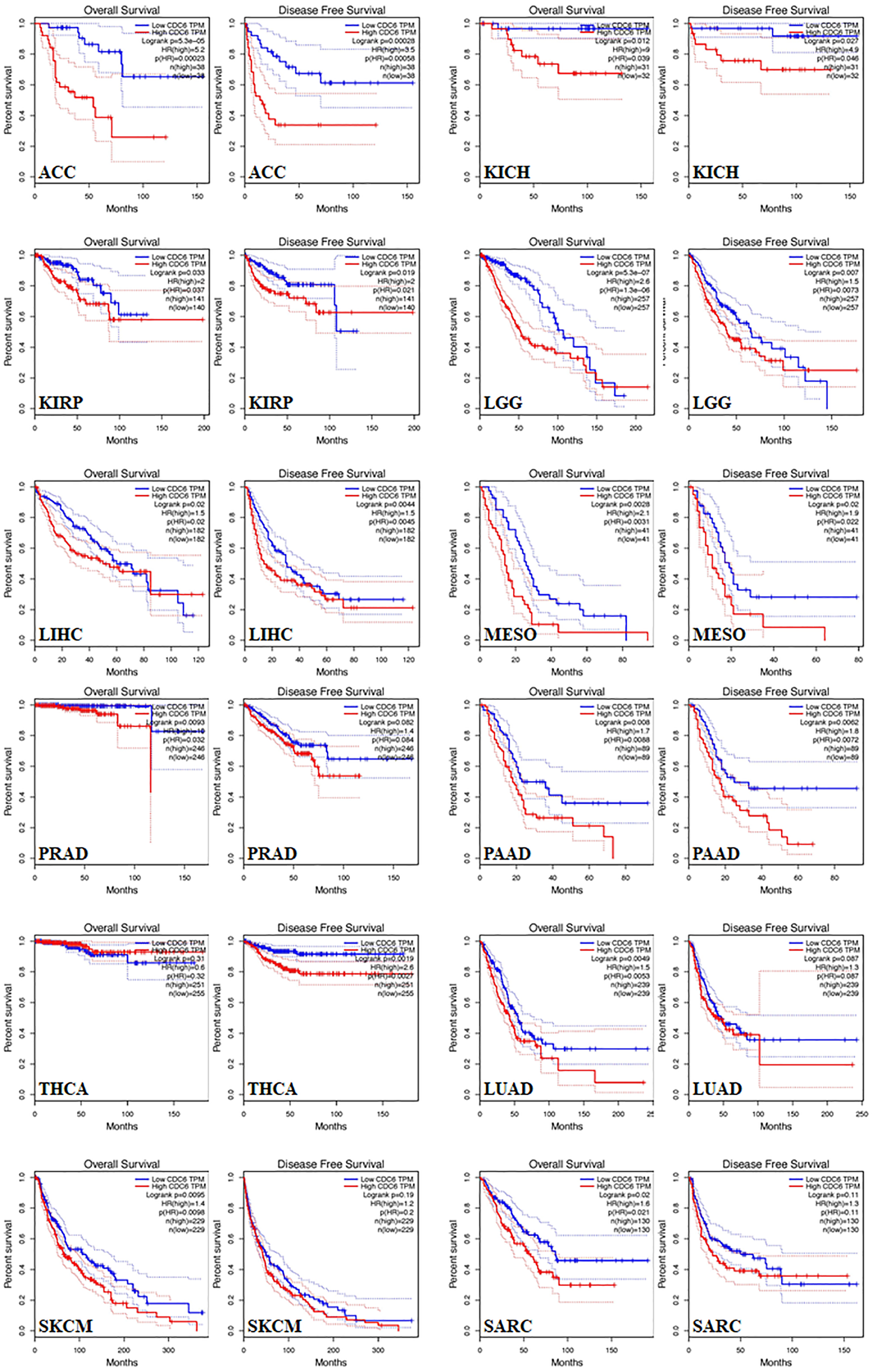


Figure S4. Overexpression of CDC6 is significantly associated with the poor overall survival and/or disease free survival of most cancer patients, related to Figure 4G. Data from TCGA and analyzed by: [**http://gepia.cancer-pku.cn/**](http://gepia.cancer-pku.cn/).
